# Combined Analysis of the Transcriptome and Metabolome Revealed the Mechanism of Petal Coloration in *Bauhinia variegata*

**DOI:** 10.3389/fpls.2022.939299

**Published:** 2022-07-12

**Authors:** Geng Zhang, Xiaohui Yang, Fang Xu, Dan Wei

**Affiliations:** Guangdong Academy of Forestry, Guangzhou, China

**Keywords:** *Bauhinia variegata*, anthocyanins, metabolome, transcriptome, structural genes, transcription factors

## Abstract

*Bauhinia variegata* petals are colorful, rich in anthocyanins, and have ornamental, nutritional, and medicinal value. However, the regulatory mechanism of anthocyanin accumulation in *B. variegata* remains unclear. In this study, a combined analysis of the metabolome and transcriptome was performed in red and white *B. variegata* cultivars in the early, middle, and blooming stages. A total of 46 different anthocyanins were identified, of which 27 showed marked differences in accumulation between the two cultivars, and contribute to their different petal colors. Malvidin 3-O-galactoside, peonidin 3-O-galactoside, cyanidin 3-O-glucoside, cyanidin 3-O-galactoside, and malvidin 3-O-glucoside were much more abundant in the second stage of flowering. In the blooming stage, except for the anthocyanins mentioned, delphinidin 3-O-galactoside and petunidin 3-O-galactoside were the most abundant anthocyanins in the red flowers, indicating that malvidin, peonidin, cyanidin, delphinidin, and petunidin were all responsible for the red color of petals in *B. variegata*. RNA sequencing identified 2,431 differentially expressed genes (DEGs), of which 26 were involved in the anthocyanin synthesis pathway. Correlations between the anthocyanin biosynthesis-related DEGs and anthocyanin contents were explored, and the DEGs involved in anthocyanin accumulation in *B. variegata* petals were identified. Eighteen of these DEGs encoded key catalytic enzymes, such as anthocyanidin reductase (ANR) and flavonoid-3′5′-hydroxylase (F3′5′H), and 17 of them encoded transcription factors (TFs) belonging to 14 families (including MYB, NAC, SPL, ERF, and CHR28). These results improve our understanding of the roles of anthocyanins, catalytic enzymes, and TFs in *B. variegata* petal-color expression.

## Introduction

Anthocyanins are important pigments that color plant tissues, and the variety in color is determined by the content and types of anthocyanins. More than 600 naturally occurring anthocyanins have been detected and isolated, most of which are classified as cyanidins, peonidins, malvidins, petunidins, pelargonidin, or delphinidins (Jin-Ming et al., 2003). In nature, anthocyanins exist as glycosides which promotes stability. Generally, pelargonidin 3-O-glucoside and cyanidin 3-O-glucoside provide red pigment to flowers and fruits, while delphinidin 3-O-glucoside is responsible for the blue color of plant organs (Harborne and Williams, 2000; Khoo et al., 2017). Different anthocyanins are responsible for the diversity of organs color among species. For example, cyanidin 3-O-(6-O-malonyl)-glucoside is the main anthocyanin responsible for the pink, red, and crimson petals of *Camellia japonica* (Fu et al., 2021). Similarly, cyanidin 3-glucoside is mainly responsible for the red flowers of *Prunus persica* (Cheng et al., 2014). Cyanidin 3-O-rutinoside, peonidin 3-O-glucoside, cyanidin 3-O-glucoside, and pelargonidin 3-O-glucoside are the dominant anthocyanins in the dark red petals of *C. japonica*, whereas cyanidin 3-O-(6-O-caffeoyl) glucoside accounts for over half of the total anthocyanins in red petals (Fu et al., 2021). In *Camellia sinensis*, the anthocyanins cyanidin O-syringic acid, petunidin 3-O-glucoside, and pelargonidin 3-O-β-d-glucoside are much more abundant in pink flowers (Zhou et al., 2020), than those of other colors. These results show that flower color can be consistent among plant species, but may also show species specificity.

Many studies have shown that the large number of anthocyanin species is driven by competition among key catalytic enzymes at certain branch points, and by the activity of a variety of regulatory factors in anthocyanin biosynthesis pathways. The biosynthesis of anthocyanins mainly depends on the flavonoid biosynthesis pathway (Shoeva et al., 2017; Nabavi et al., 2020). The biosynthesis of anthocyanin glycosides begins with the phenylpropane biosynthesis pathway, which produces chalcone from ρ-coumaroyl-CoA and 3 × malonyl-CoA under the catalysis of chalcone synthase (Sun et al., 2015). Anthocyanins are then synthesized *via* the catalysis of a series of enzymes, including chalcone flavanone isomerase, flavonoid-3′5′-hydroxylase (F3′5′H), flavonoid-3′-hydroxylase (F3′H), flavanone 3-hydroxylase, dihydroflavonol 4-reductase (DFR), anthocyanin synthase, glycosyl transferases, acyl transferase, and methyl transferase. Inactivating mutations of these enzymes’ encoding genes, especially those genes encoding key node enzymes, hinders the synthesis of anthocyanin and other flavonoids, or leads to an altered branching pathway (Spribille and Forkmann, 1982; Hemleben et al., 2004; Hoshino et al., 2009). For example, in *Gentiana scabra* and *Saintpaulia* plants, a decrease in F3′5′H expression results in a decrease in delphinidin 3-glucoside content and increase in cyanidin 3-glucoside content (Nakatsuka et al., 2006; Sato et al., 2011). However, an increase in *F3′5′H* expression significantly increased the delphinidin 3-glucoside content in transgenic *G. scabra* (Nakatsuka et al., 2006).

In addition to the above-mentioned functional genes, transcription factors (TFs) that activate or inhibit downstream genes also have important roles in anthocyanin synthesis. The main TFs that regulate anthocyanin synthesis include the MYB, bHLH, and WD40 families. The biosynthesis of most anthocyanins is regulated by a protein complex composed of these TFs (Ramsay and Glover, 2005; Tanaka and Ohmiya, 2008). Among them, MYB TFs have high tissue specificity and are generally considered to be the most critical TFs for the synthesis of plant anthocyanins. A large number of R2R3-MYB TFs have been isolated from 21 genera of plants (Yunsong et al., 2013). Most of these TFs have positive regulatory roles in anthocyanin biosynthesis, but a small number have negative regulatory roles. There is a strong correlation between the expression of the gene encoding MdMYB10 in apple (*Malus* × *domestica*) and the anthocyanin content in apple flowers and fruits, indicating that this TF is responsible for regulating anthocyanins biosynthesis in apple flowers and fruits (Espley et al., 2009). An LTR-type retrotransposon was detected in the MYB TF Ruby of blood orange (*Citrus sinensis*). This retrotransposon caused differences in the expression of *Ruby* among some blood orange varieties, and its expression level was positively correlated with the amount of delphinidin glucosides in these varieties (Butelli et al., 2012). Inhibition of PpMYB10 and ppmyb114 expression reduces anthocyanins in Chinese pear (Ni et al., 2020), and the TFs of ZjMYB5, ZjMYB113, ZjTT8, ZjGL3b, ZjGL3a, ZjWDR1, ZjWDR2, and ZjWDR3 were associated with coloration in *Ziziphus jujuba* cv. (Shi et al., 2020). In *Sophora japonica*, differential expression of the TFs SjMYB1b, SjMYBL2, SjTT8, and WD40 protein SjTTG1 may be a major driver the purple color of *S. japonica* flowers (Guo et al., 2021). In addition, members of the MYB TF family have been identified in large numbers in *Petunia*, *Arabidopsis*, and *Malus* × *domestica*, among other species, and they are significantly associated with the accumulation of plant organ pigment (Chandler and Sanchez, 2012; Zhang et al., 2019; Chen et al., 2021).

*Bauhinia variegata* is mainly distributed in southern and southwestern China and Southeast Asia. It is an important ornamental tree species owing to its unique leaves, and abundant and beautiful flowers. More than 300,000 *B. variegate* trees have been planted in Guangzhou, and there are more than 100 flower-viewing spots. Although this is an important tree species in China, study thereof has been limited. Studies of *B. variegata* have generally focused on the characteristics of tree varieties, as well as their use in gardens, cultivation, reproduction, medicines, and other practical applications. The most important ornamental trait is flower color, but the genetic factors underpinning the differential expression of flower colors have remained obscure. In this study, we compared transcriptomic and metabolic data from three developmental stages of white and dark red *B. variegate* flowers to explore the mechanisms determining flower color.

## Materials and Methods

### Plant Materials

**Figure 1:**
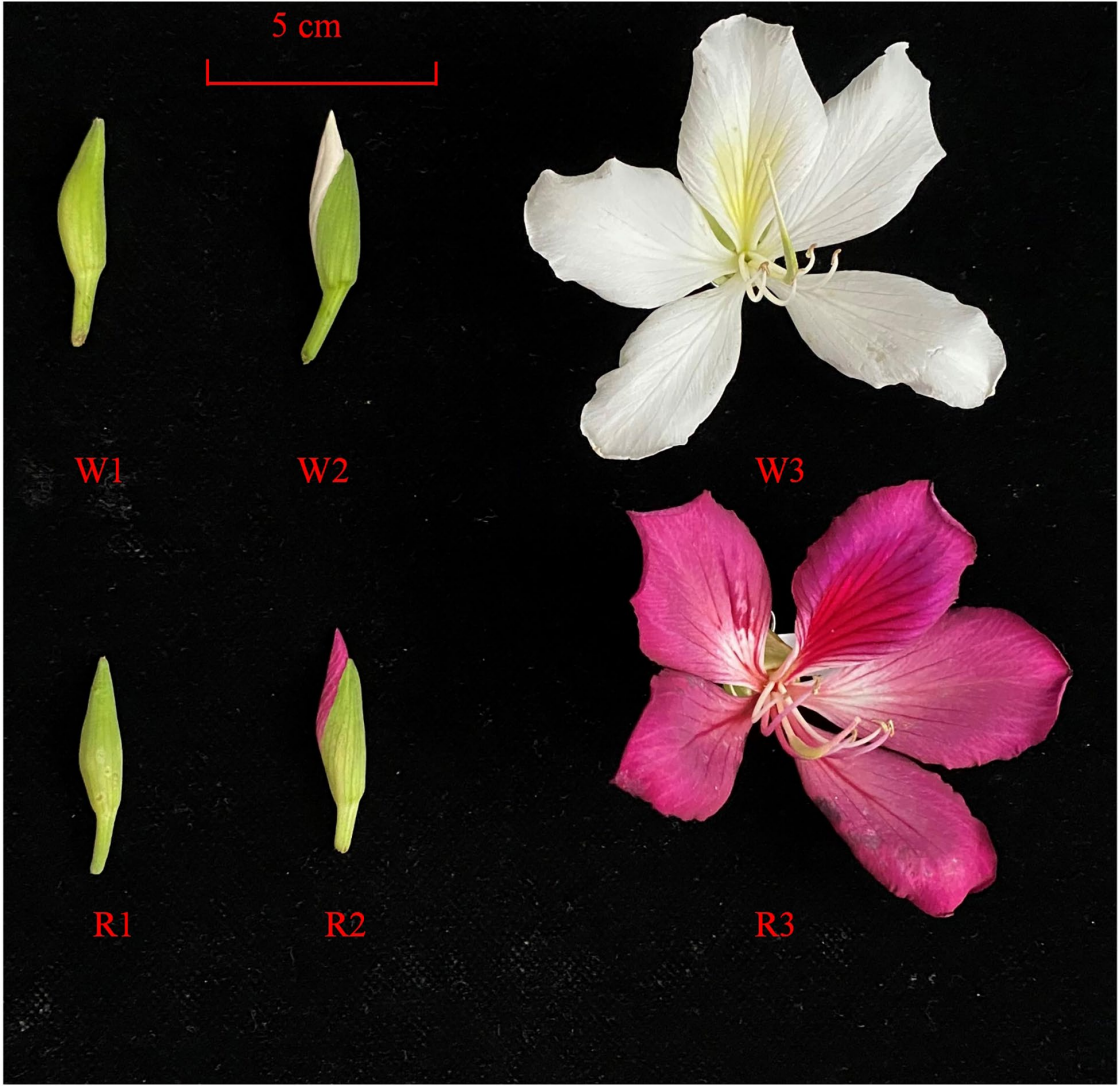
*Bauhinia variegata* in the early (W1, R1), middle (W2, R2), and blooming stages (W3, R3) in white (W) and red (R) flowers.

The materials used in this study were collected from the Germplasm Resource Garden of Guangdong Academy of Forestry (23°14′N, 113°23′E) in March 2020. Petals of the red flower (R) and white-flower (W) lines of *B. variegata* at the early stage (W1, R1), middle stage (W2, R2), and blooming stage (W3, R3) were harvested, immediately placed in a sampling tube containing liquid nitrogen, and stored in a refrigerator at −80°C for further use. Three replicates per sample were collected (Figure 1). The early stage is the bud stage with white petals, the middle stage is the bud stage with red petals in the R clone, and the blooming stage is the flower stage with opened petals.

### Metabolomic Analysis

Targeted metabolomic analyses were performed to detect anthocyanin concentrations in W and R *B. variegata* clones in different developmental stages. Three individuals were collected at each stage as biological replicates, for a total of 18 samples. The samples were quickly frozen in liquid nitrogen and stored in the refrigerator at −80°C. The samples were then ground using a mixer mill (MM 400; Retsch, Haan, Germany) with a zirconia bead for 1.5 min at 30 Hz. A total of 100 mg of powder was weighed and extracted overnight at 4°C with 1.0 ml 70% aqueous methanol containing 0.1 ml lidocaine as an internal standard. After centrifugation at 10,000 g for 10 min, the supernatant was absorbed and filtered before being subjected to liquid chromatography tandem mass spectrometry analysis. All samples were mixed as a quality control measure. The measurements and analyses were undertaken by Very Genome Co., Ltd. (Guangzhou, China). Standard procedures described previously were followed (Yuan and Grotewold, 2020).

### RNA Extraction, Quality Detection, Library Construction, and RNA Sequencing

RNA was extracted using a TRIzol kit (Invitrogen, Waltham, MA, United States) according to the manufacturer’s instructions. The Nanodrop instrument (Thermo Fisher, Waltham, MA, United States) was used to test RNA purity, and the Bioanalyzer 2,100 system (Agilent Technologies, Santa Clara, United States) was used to accurately determine the integrity of RNA. After the mRNA samples had been quantified, capture beads were used to enrich eukaryotic mRNA. Using this mRNA as a template, six-base random primers (random hexamers) were used to synthesize one-strand cDNA, followed by a two-strand cDNA synthesis reaction. DNA Clean Beads (Vazyme Biotech Co., Ltd., Nanjing, China) were then used to purify the double-stranded cDNA. The purified double-stranded cDNA was repaired, and connected to the sequencing adapter. DNA Clean Beads were used for fragment size sorting. Finally, PCR amplification was performed, and DNA Clean Beads were used to purify the PCR products and construct the final library. After the library had been constructed, Qubit 3.0 was used to determine initial concentrations, and the Agilent 2,100 Bioanalyzer was used to amplify the library insert. The ABI Step One Plus Real-Time PCR system (Applied Biosystems, Waltham, MA, United States) was used to accurately quantify the effective concentration of the libraries, which were then pooled according to their effective concentrations. The offline data volume were determined, and sequencing was performed using the Illumina HiSeq^™^ 2000 platform (Illumina, San Diego, CA, United States).

### *De novo* Assembly of Transcripts, Single Gene Expression Analysis, and Basic Annotations

A total of 18 libraries (two flower colors × three petal developmental stages × three replicates) were constructed. Clean reads were obtained by removing reads with adapters, *N* with a ratio greater than 10% of reads (indicating that base information cannot be determined), and low-quality raw reads. After obtaining clean reads, Trinity software was used to assemble the transcriptome. Standardization of single gene expression data according to transcripts per kilobase of exon model per million mapped reads (TPM) was carried out using the following formula:

TPM = A*(∑(A))^−1^*10^6^,

where A is the total reads mapped to gene*10^3^/gene length in base pairs.

BlastX and BlastN software were used to compare the assembled transcripts with functional annotations from five databases (Nr, KOG, Pfam, KEGG, and SwissProt). Nr is a comprehensive and non-redundant protein sequence database. We divided the Nt and Nr databases into animal, plant, fungi, and bacteria categories, and allocated Unigene sequences to these categories to obtain the corresponding functional annotations. Blast2GO software was used to allocate Unigene data that had previously been categorized using the Nr database to three categories in the Gene Ontology (GO) database: biological process, cellular component, and molecular function. All unigenes were aligned to the Kyoto Encyclopedia of Genes and Genomes (KEGG) database and statistically annotated at levels 1 and 2. Hmmscan software was used to annotate the Unigene sequences in the Pfam database and classify them into families according to protein domains (protein function annotation).

### Correlation Analysis of Sample Expression, Analysis of Differentially Expressed Genes

Correlation analysis of paired replicates was performed to determine the reliability of the results. The closer the correlation coefficient was to 1, the better the repeatability of two parallel experiments. RESeq2 software was used to analyze differential expression of RNAs between two different groups, and EdgeR was used to detect differences between two samples. Differentially expressed genes (DEGs) were identified using a false discovery rate < 0.05 and |log2 fold change (FC)| ≥ 1 as the threshold.

### Transcriptome Sequencing Data Accuracy Verification

To assess the accuracy of the transcriptome sequencing data, 10 key enzyme DEGs in the synthesis pathway were randomly selected for real-time quantitative PCR (qRT-PCR) verification. cDNA synthesis was performed using the FastKing RT Kit [Tiangen Biochemical Technology (Beijing) Co., Ltd., Beijing China], and Primer 6.0 was used to design specific primers (Supplementary Table S1). Using 18 s rRNA as the internal control, qRT-PCR was performed on a 7,500 RT-PCR instrument using a Real-time PCR kit (TaKaRa, Shinga, Japan) with three repetitions for each sample. The R software (version 4.0.3)^1^ was used to analyze the correlations between the qRT-PCR and transcriptome sequencing data of genes at each developmental stage, for both flower colors.

## Results

### Anthocyanin Content in the Petals of *Bauhinia variegata* During Different Periods

**Figure 2:**
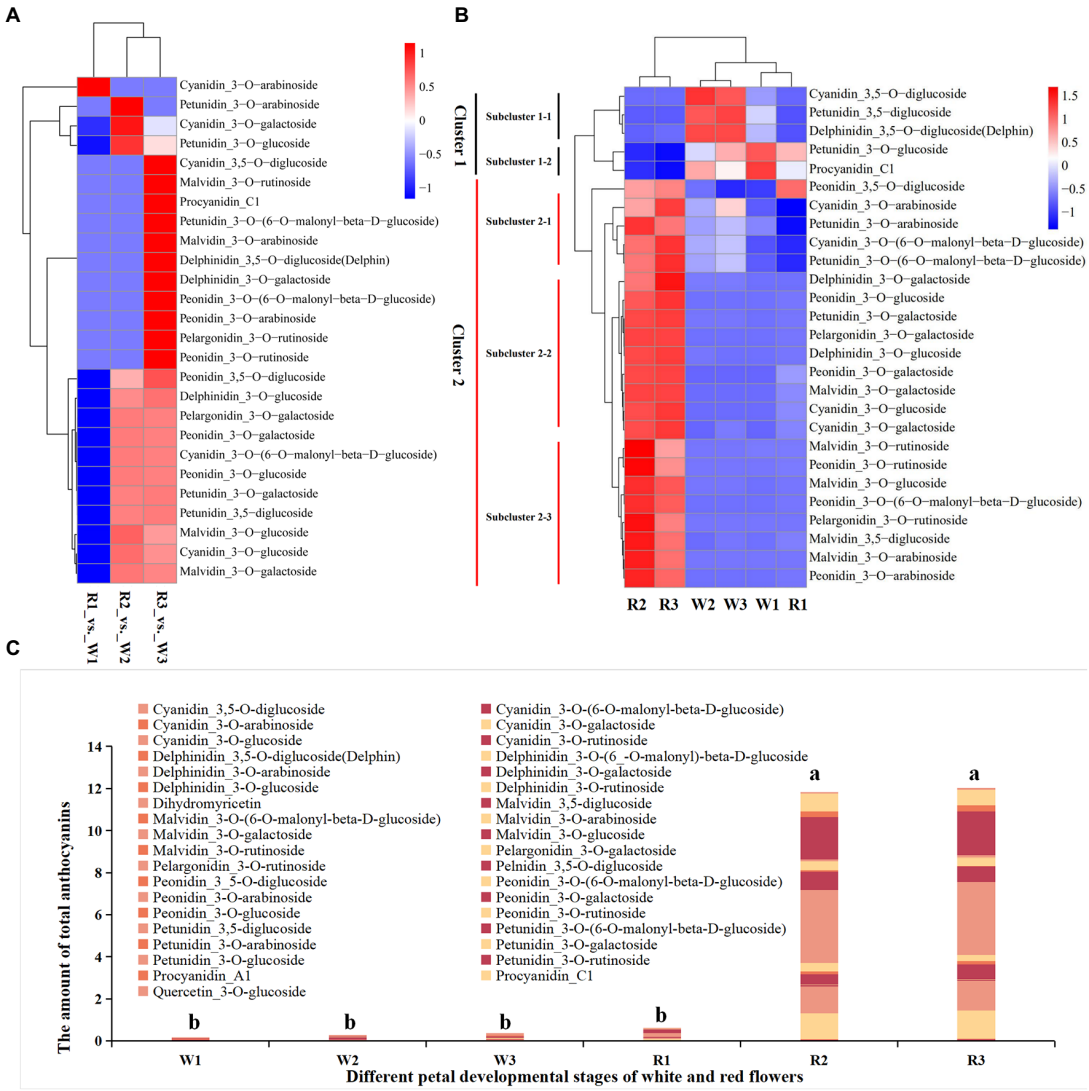
Heatmap of metabolites in the early (W1, R1), middle (W2, R2), and blooming stages (W3, R3) in white (W) and red (R) flowers. **(A)** Heatmap of differentially accumulated metabolites at different stages and fold changes. **(B)** Heatmap of differentially accumulated metabolites at different stages. The amounts of metabolites detected by an absolute quantitative method were used for heatmap clustering. **(C)** Total amount of anthocyanins at different stages of the two lines. a and b indicate significant difference (*p* < 0.05).

Forty-six types of anthocyanins were detected in petal tissues of R and W clones of *B. variegata* in the three developmental stages (Figures 2A–C; Supplementary Table S2). Metabolites were detected by an absolute quantitative detection method, and differential metabolites (DMs) were identified according to a threshold of *p* < 0.05. Twenty-seven anthocyanins were DM-enriched in the three developmental stages of the W and the R clones. All 27 metabolites were subjected to heatmap clustering (Figures 2A,B). The DMs for W1–W3 and R1 were clustered into group G1, whereas those for R2 and R3 were clustered into group G2, consistent with the color of the sampled petals. In addition, no significant difference in total anthocyanins was found among W1–W3, and R1 flower petals (Figure 2C), or between R2 and R3 in G2. However, the total amount of anthocyanins in G2 was significantly higher than in G1. Further study showed that cyanidin_3-O-arabinoside was the only DM in W1, with an FC of 0.208, when comparing R (0.00141 mg g^−1^) and W (0.00676 mg g^−1^). Significant differences in 14 DMs were found between R and W flowers, at the initial stage of petal coloration. Two cyanidins (cyanidin_3-O-glucoside, cyanidin_3-O-galactoside), two peonidins (peonidin__3-O-glucoside, peonidin_3-O-galactoside), two malvidins (malvidin_3-O-glucoside, malvidin_3-O-galactoside), one petunidin_3-O-galactoside, one pelargonidin_3-O-galactoside, and delphinidin_3-O-glucoside had FC values of 13.70–14,680.38. Absolute quantitative values ranged from 0.145698 to 3.475012 mg g^−1^, indicating that these metabolites may be the main contributors to the initial stage of petal coloration. In the late stage of the petal coloration, 25 DMs were detected; 13 of these were also identified in the comparison between R3 and W3, with an FC similar to that in the R2 vs. W2 comparison. Nine of the other 12 metabolites were much more abundant in the R flowers, with FC values of 2.16–2844.24 and absolute quantitative values of 0.00094–0.726421 mg g^−1^ in R flowers. Malvidin, cyanidin, and peonidin were also much more abundant in R2 and R3 than W2 and W3, respectively (Supplementary Figure S1). Metabolomic data showed that the red coloration in *B. variegata* petals was mainly related to the contents of malvidin, cyanidin, and peonidin of certain metabolites.

### *De novo* RNA Sequencing

To further analyze the genetic basis of *B. variegata* petal coloring, *de novo* RNA sequencing was used to compare genes that were differentially expressed between the R and W flowers at different stages. Eighteen libraries (three petal discoloration stages × two red and white flowers × three replications) were sequenced. After filtering the raw reads, high-quality clean reads for each sample were obtained (Table 1). The number of clean reads ranged from 17,134,964 to 23,467,387, and the number of clean bases ranged from 2.57 to 3.52 Gb (Table 1). GC content ranged from 44.22% to 44.91%. The Q20 and Q30 values of the clean reads ranged from 99.12% to 98.64% and 98.14% to 98.66%, respectively. After assembly, 181,335 unigenes were obtained. Using BlastX and BlastN software, the genes were mapped to seven databases: Nr, Nt, SwissProt, KEGG, KOG, Pfam, and GO (Table 2; Supplementary Figure S2A). The numbers of genes mapped to these databases ranged from 76,785 (Pfam) to 116,314 (Nr), accounting for 42.34%–64.14% of all genes. In total, 137,626 genes (75.9%) were mapped to at least one of the databases. MISA was used to identify single-strand repeats (SSRs), and different SSR lengths were detected. The most abundant SSRs were mono-nucleotide repeats (32,387 SSRs), followed by di-nucleotide repeats (13,526 SSRs). ACT/AGT were the least abundant SSRs (*n* = 62), followed by CG/CG (*n* = 87; Supplementary Figure S2B). The qRT-PCR results for 10 randomly selected genes were significantly correlated with the transcriptomic profiling data (Supplementary Figure S3, R^2^ = 0.91), indicating that the transcriptomic profiling data were sufficiently accurate for further study.

**Table 1:** Summary of sequencing data.

Samples	Clean reads	Base (bp)	Basesize (GB)	GC (%)	Q20(%)	Q30(%)
W1-1	20,403,493	3,060,523,950	3.060524	44.22	99.43	98.24
W1-2	18,849,901	2,827,485,150	2.827485	44.31	99.22	98.22
W1-3	18,109,970	2,716,495,500	2.716496	44.66	99.23	98.17
W3-1	19,255,783	2,888,367,450	2.888367	44.43	99.31	98.21
W3-2	19,142,879	2,871,431,850	2.871432	44.62	99.55	98.63
W3-3	21,084,021	3,162,603,150	3.162603	44.53	99.24	98.27
W4-1	19,178,062	2,876,709,300	2.876709	44.78	99.25	98.15
W4-2	21,526,502	3,228,975,300	3.228975	44.91	99.30	98.26
W4-3	19,193,339	2,879,000,850	2.879001	44.78	99.37	98.66
R1-1	22,468,051	3,370,207,650	3.370208	44.85	99.36	98.21
R1-2	23,467,387	3,520,108,050	3.520108	44.38	99.41	98.20
R1-3	19,246,305	2,886,945,750	2.886946	44.27	99.34	98.16
R2-1	20,316,514	3,047,477,100	3.047477	44.32	99.37	98.21
R2-2	21,494,214	3,224,132,100	3.224132	44.31	99.12	98.30
R2-3	20,384,880	3,057,732,000	3.057732	44.45	99.38	98.14
R3-1	20,346,203	3,051,930,450	3.05193	44.23	99.49	98.16
R3-2	17,134,964	2,570,244,600	2.570245	44.35	99.64	98.15
R3-3	20,485,124	3,072,768,600	3.072769	44.57	99.52	98.24

W indicates white flower; R indicates red flower. W1 and R1 represent the first stages of white and red flowers, respectively; W2 and R2 represent the second stages of white and red flowers, respectively, and W3 and R3 represent the third stages of white and red flowers, respectively.

**Table 2:** Annotation of transcripts in the NR, NT, GO, KOG, Pfam, KEGG, and SwissProt databases.

Annotation	Total	Nr	Nt	SwissProt	KEGG	KOG	Pfam	GO	Overall
Number	181,335	116,314	110,004	86,132	94,049	97,689	76,785	83,628	137,626
Percentage/%	100	64.14	60.66	47.50	51.86	53.87	42.34	46.12	75.90

**Table 3:** Transcription factors significantly correlated with cyanidin, malvidin, and peonidin.

Gene ID	Transcription factors	Cyanidin	Malvidin	Peonidin
TRINITY_DN1414_c0_g1_i19	SPL	0.96^**^	0.95^**^	0.97^**^
TRINITY_DN10500_c0_g1_i15	CHR28	0.94^**^	0.93^**^	0.96^**^
TRINITY_DN4590_c0_g2_i1	MYB	0.92^**^	0.91^**^	0.94^**^
TRINITY_DN2760_c0_g1_i1	ERF	0.89^**^	0.90^**^	0.90^**^
TRINITY_DN1414_c0_g1_i18	SPL	0.87^**^	0.86^**^	0.90^**^
cluster_contig5226	TCP	0.86^**^	0.86^**^	0.86^**^
TRINITY_DN592_c0_g2_i1	DRE1A	0.84^*^	0.86^**^	0.83^*^
TRINITY_DN23949_c0_g1_i4	NAC	0.79^*^	0.79^*^	0.82^*^
cluster_contig4934	TCP	0.78^*^	0.78^*^	0.76^*^
TRINITY_DN592_c0_g2_i2	DRE1A	0.75^*^	0.76^*^	0.74^*^
TRINITY_DN3286_c0_g1_i12	MYB_C	0.74^*^	0.74^*^	0.75^*^
TRINITY_DN8671_c0_g2_i8	TF	0.72^*^	0.74^*^	0.70^*^
TRINITY_DN784_c0_g2_i7	GATA	0.72^*^	0.73^*^	0.73^*^
TRINITY_DN3021_c0_g1_i8	B10	0.71^*^	0.71^*^	0.73^*^
TRINITY_DN12725_c0_g1_i1	VIP	0.70^*^	0.71^*^	0.72^*^
TRINITY_DN5417_c0_g1_i17	Myb_like	−0.69^*^	−0.70^*^	−0.71^*^
TRINITY_DN1849_c0_g1_i5	MEIS	−0.93^**^	−0.93^**^	−0.93^**^

* *p* < 0.05;

** *p* < 0.01.

### Identification of DEGs Between R and W Flowers of *Bauhinia variegata* in Different Developmental Stages

**Figure 3:**
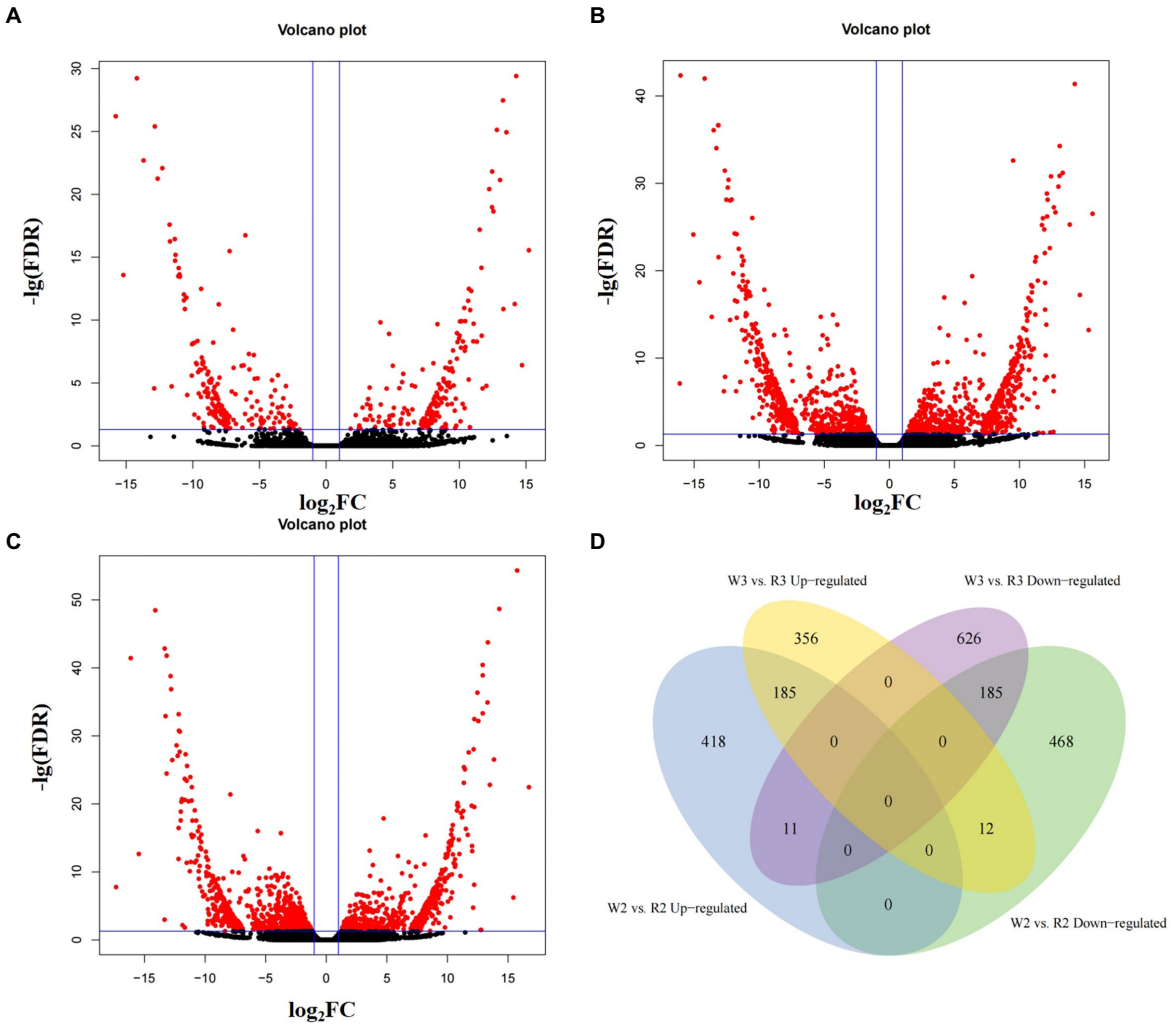
Volcano plot and Venn diagram of differentially expressed genes at different stages between white (W) and red (R) flowers. **(A)** Early stage (W1, R1); **(B)** middle stage (W2, R2); **(C)** blooming stage (W3, R3); and **(D)** venn analysis of up- and down-regulated differentially expressed genes at middle and blooming stages.

The TPM of every transcript was calculated to normalize gene expression levels of within and among samples. After filtering out genes with TPM < 0.1 in all samples, the remaining genes were used to identify DEGs among different developmental stages. In the comparison of W1 and R1, 410 DEGs were obtained, including 203 that were upregulated and 207 that were downregulated (*Q* < 0.05, Figures 3A–C). In the comparison of W2 and R2, 614 DEGs were upregulated and 665 were downregulated. In the comparison of W3 and R3, 1,375 DEGs were obtained, including 553 that were upregulated in W flowers and 822 that were upregulated in R flowers. As the petals started to show color differentiation during the middle stage, the DEGs for W2 vs. R2 and W3 vs. R3 were compared to identify common DEGs that may be responsible for petal discoloration (Figure 3D). In total, 185 DEGs were downregulated in the comparisons of W2 and R2 and W3 and R3, indicating that these DEGs may be responsible for the petal discoloration at different stages. The other 468 and 626 DEGs were uniquely enriched in the comparisons of W2 and R2, and W3 and R3, indicating a role in petal discoloration at different stages of development.

### Functional Annotation of DEGs

**Figure 4:**
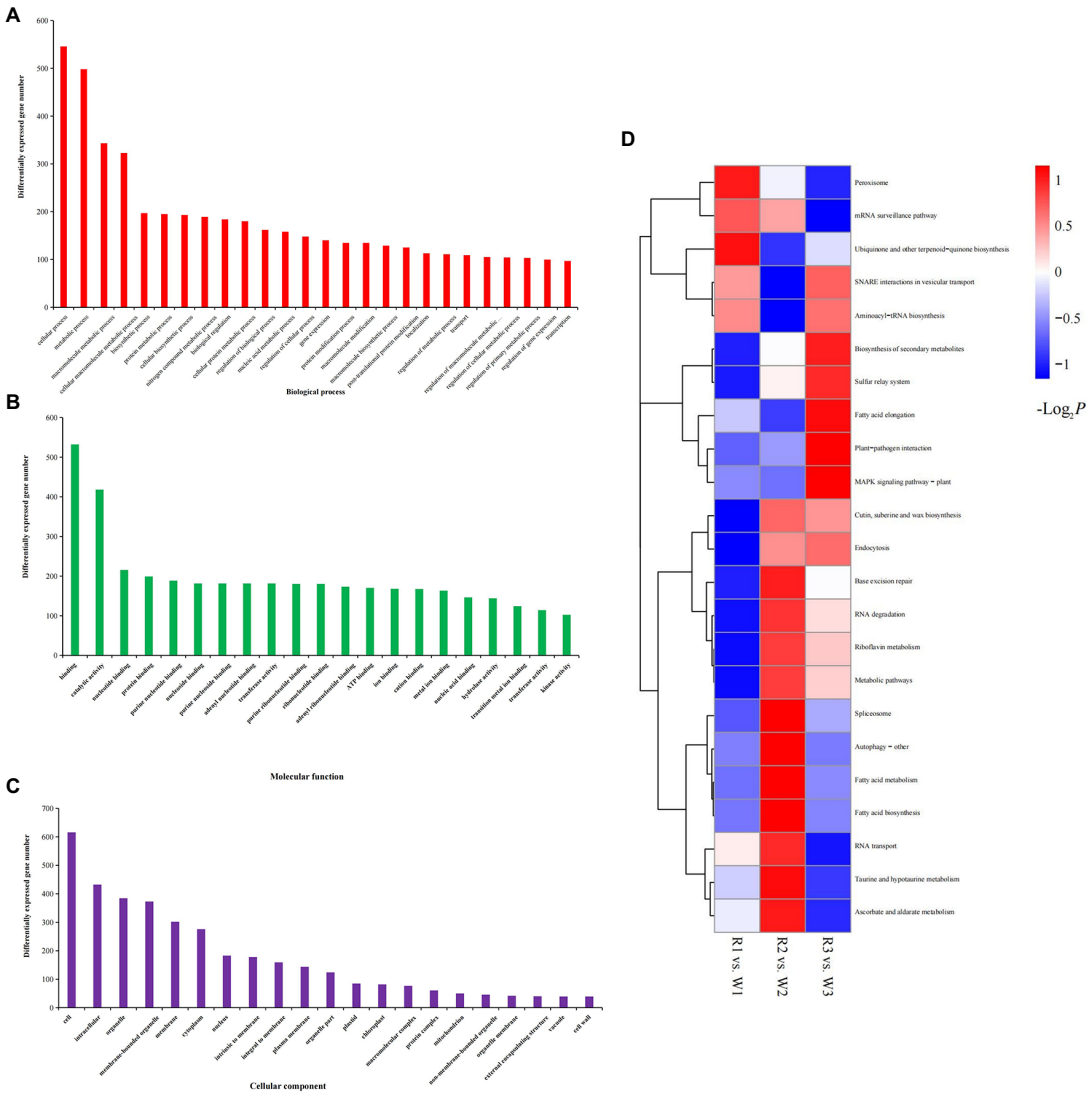
Gene Ontology analysis and Kyoto Encyclopedia of Genes and Genomes (KEGG) enrichment of differentially expressed genes (DEGs) identified by comparison of white (W) and red (R) flowers at early (W1, R1), middle (W2, R2), and blooming (W3, R3) stages. **(A)** DEGs enriched in biological process; **(B)** DEGs enriched in cellular components; and **(C)** DEGs enriched in molecular functions; and **(D)** KEGG enrichment analysis of DEGs.

To explore their possible functions, all DEGs were mapped to the GO database. Most DEGs were enriched in cellular (*n* = 546; 22.45%), metabolic (*n* = 498; 20.48%), and macromolecule metabolic (*n* = 343; 14.10%) processes in the biological process category. Their molecular functions included binding (*n* = 532; 21.88%), catalytic activity (*n* = 418; 17.19%), transferase activity (*n* = 181; 7.44%), and nucleic acid binding (*n* = 146; 6.00%). In the cellular component category, more than 25.33% of the DEGs were enriched in cells or cell parts, and more than 15.79% were enriched in intracellular parts, organelles, and membrane-bound organelles (Figures 4A–C). The 185 DEGs common to W2 vs. R2 and W3 vs. R3 comparisons were significantly enriched in 23 molecular functional categories. Twenty-two of these functional categories were related to the binding activities of transition metals, proteins, protein binding and purine ribonucleoside triphosphate binding; the other one was catalytic activity, indicating the role of this group of DEGs in flower petal discoloration (Supplementary Table S3). The KEGG annotation showed that all DEGs from the various petal developmental stages of W and R flowers could be mapped to 23 KEGG pathways (Figure 4D). Among these pathways, DEGs involved in the biosynthesis of secondary metabolites, cutin, suberin, peroxisome, and wax, and in metabolic pathways, were significantly enriched in all three comparisons. DEGs involved in ascorbate and aldarate metabolism, taurine and hypotaurine metabolism, and riboflavin metabolism were significantly enriched in the W2 vs. R2 comparison, while DEGs involved in plant-pathogen interaction, MAPK signaling pathway-plant, and fatty acid elongation were significantly enriched in the W3 vs. R3 comparison.

### DEGs Involved in the Anthocyanin Biosynthesis Pathway

**Figure 5:**
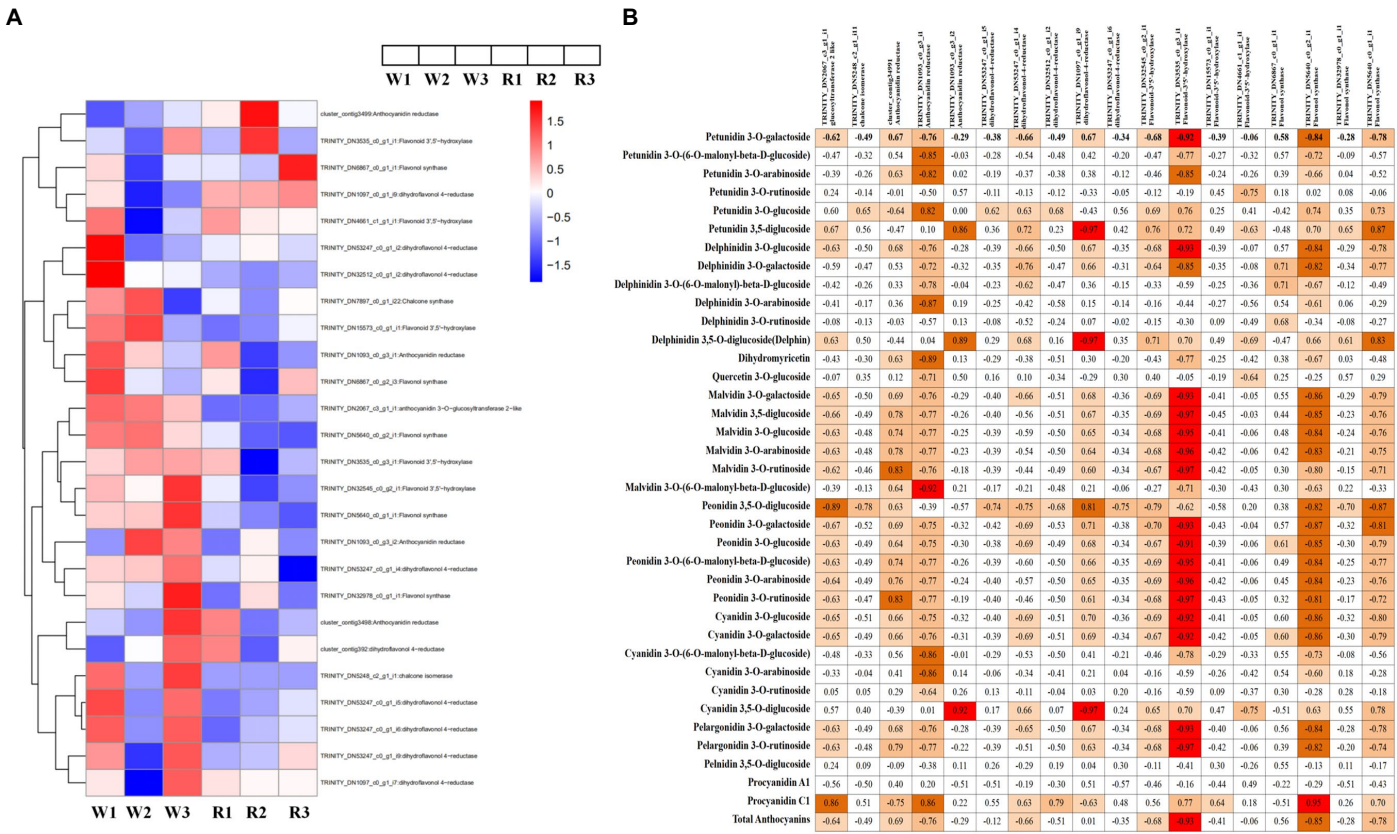
Heatmap of genes encoding key enzymes in the anthocyanin biosynthetic pathway and correlation analysis between anthocyanin content and expression levels of key anthocyanin-biosynthetic genes. **(A)** Heatmap of structural genes encoding key enzymes in the anthocyanin biosynthetic pathway. Expression level data of different enzyme-coding genes were used in the heatmap analysis. Red indicates higher expression levels and blue indicates lower expression levels. **(B)** Correlation analysis between anthocyanin content and the expression levels of key anthocyanin-biosynthetic genes.

To explore the differences in anthocyanin biosynthesis between petals from the W and R clones, DEGs in the anthocyanin synthesis pathway were identified. In total, 26 DEGs were enriched in the anthocyanin synthesis pathway, including one DEG each encoding chalcone synthase, anthocyanidin 3-O-glucosyltransferase 2-like, and chalcone isomerase. Also, there were four DEGs encoding anthocyanidin reductase (ANR), nine encoding DFR, five encoding flavonoid-3′5′-hydroxylase (F3′5′H), and five encoding flavonol synthase (Figure 5A). Pearson correlation analysis of the expression levels of these DEGs and DM contents showed that 18 DEGs were significantly correlated with the contents of at least one anthocyanin, either positively or negatively (Figure 5B). Among these, the DEGs encoding ANR (TRINITY_DN1093_c0_g3_i1) was positively correlated with procyanidin C1 (*r* = 0.86, *p* < 0.001) and negatively correlated with 27 metabolite, with *r* values ranging from −0.92 to −0.64 (*p* < 0.001). Significantly correlated metabolites included three petunidins, four delphinidins, two flavonoids, six malvidins, two pelargonidin, five peonidins and five cyanidins. Two DEGs (TRINITY_DN32545_c0_g2_i1 and TRINITY_DN3535_c0_g3_i1) encoding F3′5′H were significantly positively correlated with delphinidin 3,5-O-diglucoside, with r values of 0.71 and 0.70, respectively (*p* < 0.001). These DEGs were also positively correlated with petunidin 3-O-glucoside (*r* = 0.69 and 0.76, respectively) and petunidin 3,5-diglucoside (*r* = 0.76 and 0.72, respectively). A DEG (TRINITY_DN53247_c0_g1_i4) encoding DFR was significantly positively correlated with four metabolites (*r* > 0.63, *p* < 0.001), i.e., petunidin 3-O-glucoside, cyanidin 3,5-O-diglucoside, delphinidin 3,5-O-diglucoside, and petunidin 3,5-diglucoside. TRINITY_DN53247_c0_g1_i4 was also strongly correlated with total anthocyanins (*r* = 0.68, *p* < 0.05).

### Detection of TFs Contributing to Red Flower Color

TFs are important regulators of various plant growth and development processes, as well as in the coloration of plant tissues. In our study, 99 differentially expressed TFs were identified, most of which belonged to the ERF, WRKY, ARF, MADS, bHLH, TCP, RMA, MYB, NAC, SPL, and bZIP families Supplementary Tables S5, S6). These differentially expressed TFs were visualized based on their TPM values (Supplementary Figure S3). Eighty-one TFs belonging to 35 TF families were significantly correlated with at least one anthocyanin (*r* ≥ 0.6, *p* < 0.01). To further analyze TFs associated with flower color variation, we performed a correlation analysis between TFs and the three classes of anthocyanins (cyanidin, malvidin and peonidin) that differed most between the W and R clones (Table 3); Seventeen TFs were significantly correlated with the three kinds of anthocyanins, 15 of them positively correlated. For example, TRINITY_DN4590_c0_g2_i1, which encoded the TF MYB, was positively correlated with cyanidin, malvidin and peonidin (*r* = 0.92, 0.91 and 0.94, respectively). This DEG was also positively and negatively correlated with 23 and five anthocyanins, respectively. Two DEGs (TRINITY_DN1414_c0_g1_i19 and TRINITY_DN1414_c0_g1_i18) encoding SPL, two (TRINITY_DN592_c0_g2_i2 and TRINITY_DN592_c0_g2_i1) encoding DRE1A, one (TRINITY_DN3286_c0_g1_i12) encoding MYB-C, one DEG (TRINITY_DN2760_c0_g1_i1) encoding ERF, and one (TRINITY_DN10500_c0_g1_i15) encoding CHR28 had similar correlations to those of MYB, indicating that these genes may be promote flower coloration. TRINITY_DN5417_c0_g1_i17, which encoded an MYB-like TF and TRINITY_DN1849_c0_g1_i5, which encoded MEIS, were both negatively correlated with the three kinds of anthocyanins, indicating that these genes may suppress flower coloration.

## Discussion

### Identification of Anthocyanin in *Bauhinia variegata* Petals

Anthocyanins are a group of metabolites involved in the coloration of various plant organs, including flowers, fruits, and tubers (Tang et al., 2020; Zhang et al., 2020). There are more than 600 species of anthocyanins in nature, most of which are derived from six well-known anthocyanins: cyanidin, delphinidin, pelargonidin, peonidin, malvidin, and petunidin (proportion of 50%, 12%, 12%, 12%, 7%, and 7%, respectively; Castañeda-Ovando et al., 2009; Jaakola, 2013).

The molecular structures of these anthocyanins, and their functions in plant organs, have been studied. Generally, cyanidin appears reddish-purple (magenta), while delphinidin is responsible for blue-reddish and purple colors, pelargonidin for red color, pelargonidin for orange color, and peonidin for magenta color (Robinson and Robinson, 1932). Malvidin is abundant in blue-colored flowers, while petunidin has been detected in purple flower petals (Tanaka and Ohmiya, 2008). In our study, different anthocyanins were detected in different developmental stages of white flowers and red flower petals. Twenty-seven anthocyanins were identified as DMs enriched in the early, middle, and blooming stages of flower development. In the initial stage of petal discoloration, malvidin 3-O-galactoside, peonidin 3-O-galactoside, cyanidin 3-O-glucoside, cyanidin 3-O-galactoside, and malvidin 3-O-glucoside were the most abundant anthocyanins in R flowers, indicating that these anthocyanins may be responsible for the red petals seen in the second stage of flowering. The amounts of these anthocyanins were consistent with those in the third stage, except in the case of delphinidin 3-O-galactoside and petunidin 3-O-galactosid. Our results indicate that malvidin, peonidin, and cyanidin are responsible for the red petals of *B. variegata*. These findings differ from those of some previous studies. For example, Fu et al. (2021) reported that cyanidin, pelargonidin, delphinidin, and peonidin (but not malvidin and petunidin) had important functions in *C. japonica* petals (Fu et al., 2021). Xue et al. (2019) found that cyanidin, pelargonidin, and delphinidin were the main anthocyanins in the red flower petals of strawberries, whereas in our study, malvidin was the most abundant anthocyanin in red petals.

Anthocyanins in organisms usually exist in the form of glycosides, thus maintaining stability (Ongkowijoyo et al., 2018; Loarce et al., 2021). In our study, five types of glycoside were detected, including three monoglycosides (galactoside, glucose, and arabinose) and two polyglycosides (di-glucoside and rutinose). Monoglycosides accounted for 99.99%and 97.89% of the total anthocyanins in R2 and R3, respectively, whereas 74.13% and 71.86% of total anthocyanins were in the form of galactosides in *B. variegata* petals. Our results were consistent with those of a previous study showing that more than 94% of the anthocyanins in strawberries were monoglycosides (Kelebek and Selli, 2011). However, our results differed from studies showing that anthocyanins (as syringic acid) were abundant in the rubellis mutant tepals of *Michelia maudiae*. This result is consistent with the findings of Fu et al. (2021), who reported that 3.25% of total anthocyanins were cyanidin-O-syringic acid (Lang et al., 2019).

Genetic differences among species may underlie the diverse array of anthocyanins, both in terms of contents and composition. These differences are key factors contributing to flower color variation. Generally, darker flowers result from higher anthocyanin contents in petals. In *Gentiana triflora*, much higher levels of anthocyanins were detected in blue than white flowers, whereas no anthocyanins were detected in white flowers (Nakatsuka et al., 2008). These findings support the results of our study showing that the total anthocyanin content was much higher in R2 and R3 compared with R1, in all three W stages (Figure 1). In *C. japonica*, 11 anthocyanins were found to be DMs in different-colored petals. Cyanidin 3-O-(6-O-malonyl) was the main driver of the pink, red, and crimson petal phenotypes (Fu et al., 2021). These results were consistent with Cheng et al. (2014), who concluded that cyanidin 3-glucoside was the primary component in the red flowers of *P. persica*. Cheng et al. (2014) drew similar conclusions to Fu et al. (2021), who found that cyanidin 3-O-rutinoside, peonidin 3-O-glucoside, cyanidin 3-O-glucoside, and pelargonidin 3-O-glucoside play key roles in deepening the color of *C. japonica* petals. These authors proposed that cyanidin O-syringic acid, petunidin 3-O-glucoside, and pelargonidin 3-O-β-d-glucoside accumulate in pink flowers, implying that they may have play an important role in the coloration of pink flowers in *C. sinensis*. In our study, however, malvidin 3-O-galactoside, peonidin 3-O-galactoside, cyanidin 3-O-glucoside, and cyanidin 3-O-galactoside were the most abundant anthocyanins in R2 and R3, but not in white flowers or R1, indicating that these four anthocyanins may contribute to the red color of *B. variegata* flowers.

### Key Structural Genes Responsible for Anthocyanin Synthesis in *Bauhinia variegata* Petals

To identify the DEGs potentially making the greatest contributions to petal-color variation, GO and KEGG analyses were performed. The GO results showed that the DEGs were enriched in cellular, metabolic, and macromolecule metabolic processes, as well as the biosynthesis of secondary metabolites and fatty acid metabolism. These results are consistent with ones showing that DEGs involved in these metabolic pathways may be important determinants of tissue color in mutant *Z. jujuba* cv. (Shi et al., 2020).

Flower color depends on the types and content of anthocyanins, and anthocyanin biosynthesis is controlled by competition among key enzymes, as well as enzyme substrate specificity (Holton and Cornish, 1995). Several studies have investigated the possible functions of key anthocyanin biosynthesis genes. In *Paeonia. suffruticosa*, mutation of *PsF3′H* promotes the expression of *PsFLS*, leading to differences in anthocyanin content and acyanic flowers (Gu et al., 2019). In *Gentiana*, insertion of different transposons into the *F3′5′H* sequence caused in a decrease in transcription levels of *F3′5′H*, resulting in conversion to the cyanidin pathway (Nakatsuka et al., 2006). In *Petunia hybrida*, increased expression of F3′5′H contributed to a higher delphinidin content. In our study, two F3′5′H-encoding DEGs were positively correlated with delphinidin 3,5-O-diglucoside, petunidin 3-O-glucoside, and petunidin 3,5-diglucoside. These results were consistent with the finding that F3′5′H is a key enzyme whose competitive expression is associated with a delphinidin branching pathway (Tsuda et al., 2004). In *Eustoma grandiflorum* Grise, the antisense FLS transgenic plants of a purple-flowered line produced more red flowers than the original line, and showed red buds and deeply colored pistils (Nielsen et al., 2002; Davies et al., 2003). In the anthocyanin biosynthesis pathway, FLS and DFR are important enzymes that produce colorless flavonols and colored anthocyanins in the Mitchell line of *Petunia*. Promotion of DFR and inhibition of FLS expression led to higher anthocyanin content and pink petals, and the highest anthocyanin levels were observed in the DFR-sense and FLS-antisense transgenic lines (Davies et al., 2003; Tsuda et al., 2004). In our study, the DEG encoding DFR (TRINITY_DN53247_c0_g1_i4) was positively correlated with petunidin 3-O-glucoside, cyanidin 3,5-O-diglucoside, delphinidin 3,5-O-diglucoside, petunidin 3,5-diglucoside, and total anthocyanin contents, indicating a role for this DEG in petal coloration.

### TFs Involved in Anthocyanin Synthesis in *Bauhinia variegata* Petals

TFs are groups of proteins that participate in many plant growth processes by activating or inhibiting downstream genes. Previous studies showed that TFs play important roles in plant coloration. MYB, bHLH, and WD40 are the main TFs that regulate anthocyanin biosynthesis. These TFs usually form an MYB–bHLH–WD40 protein complex that binds to the promoter regions of synthesis enzymes (Zhang et al., 2014). In *P. hybrida*, the AN11(WD40)–AN1(bHLH)–AN2(R2R3-MYB) transcription complex regulates anthocyanin synthesis in petals (Quattrocchio et al., 2006). In our study, five DEGs encoding MYB TFs and four DEGs encoding bHLH TFs were identified. The expression of TRINITY_DN4590_c0_g2_i1, which encodes MYB44, was positively correlated with total anthocyanin content (*r* = 0.907, *p* < 0.01) and the contents of 23 types of anthocyanins (*r* > 0.6, *p* < 0.01), indicating a role of MYB44 in petal coloration. Two other DEGs, TRINITY_DN749_c1_g1_i7 (encoding SPL2) and TRINITY_DN23949_c0_g1_i4 (encoding NAC35) were negatively and positively correlated, respectively, with 23 DMs and with total anthocyanin content. In *P. persica*, PpSPL1 positively regulates anthocyanin accumulation by repressing the expression of PpNAC1, which can activate the transcription of PpMYB10.1 and then promote anthocyanin biosynthesis (Zhou et al., 2015).

Different TF family members may have similar functions in different plant species. For example, TRINITY_DN1414_c0_g1_i19, which encodes SPL12, was positively correlated with most of the 37 DMs, and with total anthocyanin content, indicating that different members of SPL families have contrasting functions in anthocyanin biosynthesis. The DEGs encoding ERF78 (TRINITY_DN2760_c0_g1_i1) were positively correlated with total anthocyanin content, cyanidin, malvidin and peonidin (*r* > 0.89, *p* < 0.01). These findings were consistent with those of Fu et al. (2021), who reported that these DEGs were positive TFs in of anthocyanin accumulation (Fu et al., 2021).

## Conclusion

In this study, combined analyses of metabolome and transcriptome data were used to detect genes encoding key catalytic enzymes related to anthocyanin biosynthesis and TFs responsible for *B. variegata* petal coloration. A total of 46 anthocyanins were detected in *B. variegata* flowers, of which 27 were differentially expressed between W and R flowers. Malvidin 3-O-galactoside, peonidin 3-O-galactoside, cyanidin 3-O-glucoside, cyanidin 3-O-galactoside, malvidin 3-O-glucoside, delphinidin 3-O-galactoside, and petunidin 3-O-galactoside were shown to be responsible for the red color of the *B. variegata* petals. Moreover, 26 DEGs (in particular, ANR and F3′5′H) involved in anthocyanin biosynthesis, and 17 TFs belonging to 14 TF families (including MYB, NAC, SPL, ERF, CHR28), were identified. This study provides important insight into the role of anthocyanins in color expression in *B. variegata* petals.

## Data Availability Statement

The datasets presented in this study can be found in online repositories. The names of the repository/repositories and accession number(s) can be found at: BIG Sub (https://ngdc.cncb.ac.cn/gsub), under the accession number of CRA007203 (https://bigd.big.ac.cn/gsa/browse/CRA007203).

## Author Contributions

DW, XY, and FX conceived and designed the experiments. GZ and XY performed the experiments, analyzed the data, and wrote the manuscript. All authors contributed to the article and approved the submitted version.

## Funding

The research was supported by “the Guangzhou Science and Technology Plan Project (201904010397).”

## Conflict of Interest

The authors declare that the research was conducted in the absence of any commercial or financial relationships that could be construed as a potential conflict of interest.

## Publisher’s Note

All claims expressed in this article are solely those of the authors and do not necessarily represent those of their affiliated organizations, or those of the publisher, the editors and the reviewers. Any product that may be evaluated in this article, or claim that may be made by its manufacturer, is not guaranteed or endorsed by the publisher.

## Footnotes

^1^ http://cran.rproject.org/
