# Supplementary material for: Combined Analysis of the Transcriptome and Metabolome Revealed the Mechanism of Petal Coloration in Bauhinia variegata
Source: Front Plant Sci. 2022 Jul 12;13:939299. doi: 10.3389/fpls.2022.939299 (PMC9315375; doi:10.3389/fpls.2022.939299)
Supplement: Supplementary file 7 [file Image_1.pdf]

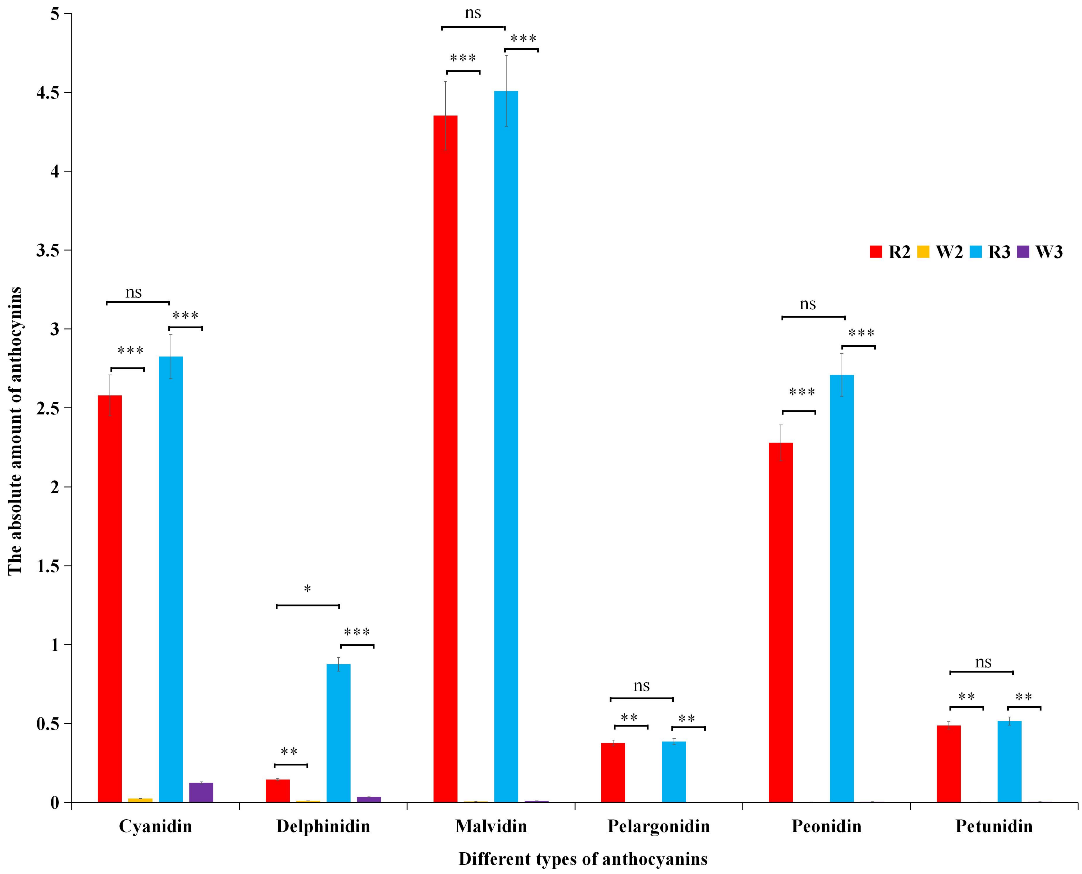

Figure S1. The amount of different anthocyanins at the second and third stage of the red (R) and white (W) flowers. * indicates $P < 0.05$; ** indicates $P < 0.01$; *** indicates $P < 0.001$.

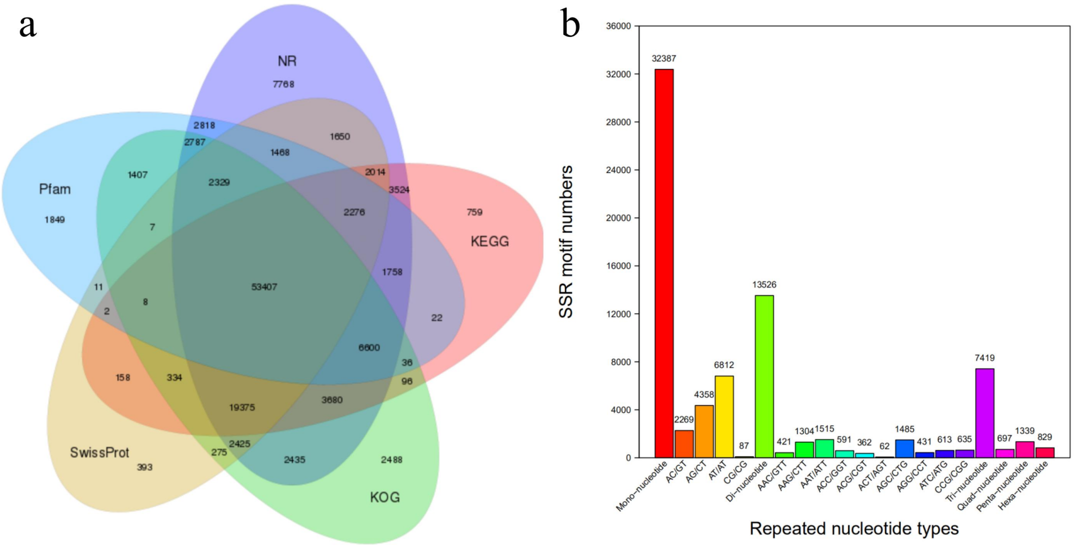

Figure S2 Annotation of transcripts using the Nr, Nt, GO, KOG, Pfam, KEGG, and SwissProt databases (a) and distribution of SSR (b).

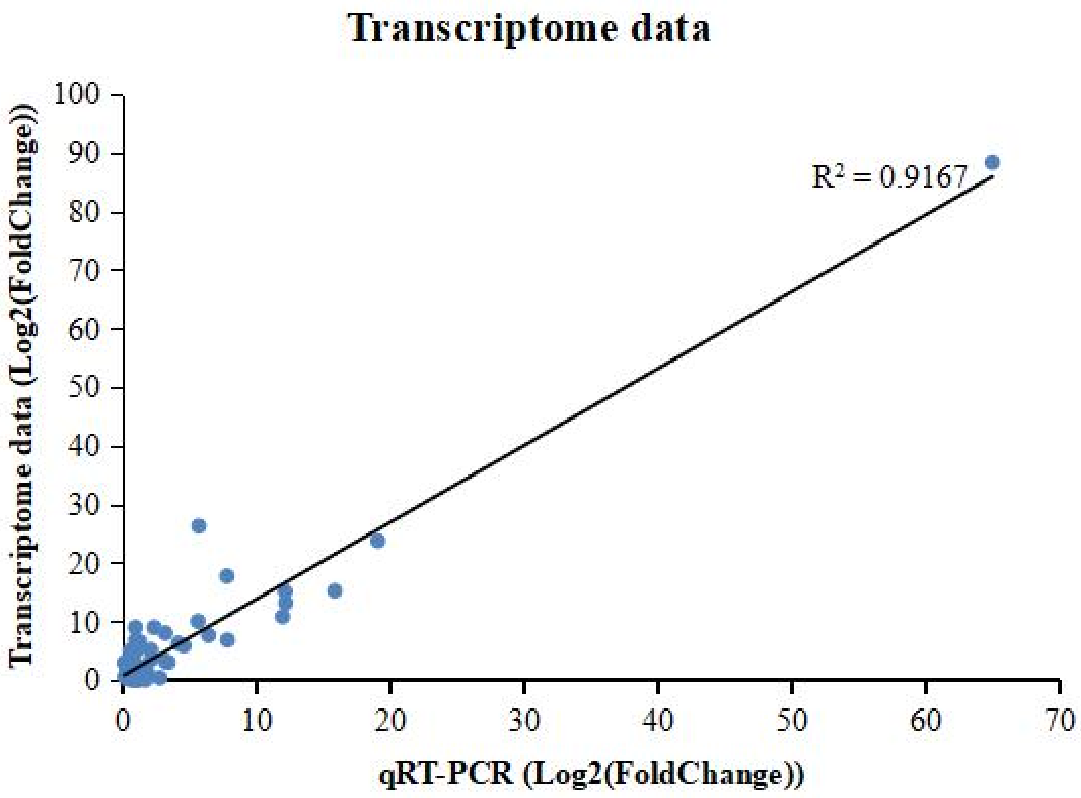

Figure S3 Transcriptome data validation by qRT-PCR.

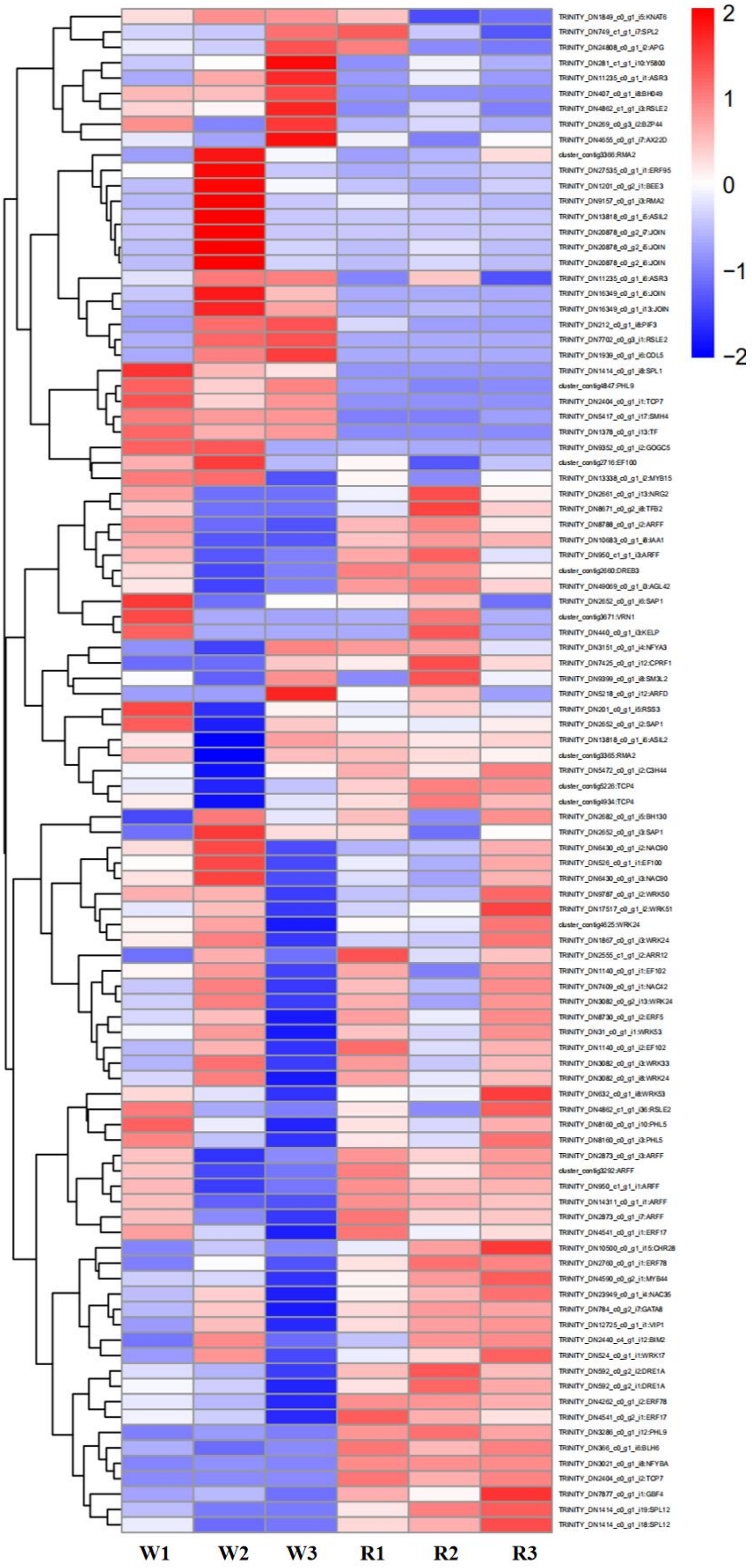

Figure S4. Heatmap of transcription factors. The expression levels of different transcription factors were used for the heatmap analysis. Red indicates higher expression levels and blue indicates lower expression levels.
